# Incidence of sodium–glucose cotransporter-2 inhibitor-associated perioperative ketoacidosis in surgical patients: a prospective cohort study

**DOI:** 10.1007/s00540-024-03335-3

**Published:** 2024-03-17

**Authors:** Hiroyuki Seki, Norifumi Kuratani, Toshiya Shiga, Yudai Iwasaki, Kanae Karita, Kazuki Yasuda, Natsuko Yamamoto, Yuko Nakanishi, Kenji Shigematsu, Kensuke Kobayashi, Junichi Saito, Ichiro Kondo, Nozomu Yaida, Hidenobu Watanabe, Midoriko Higashi, Tetsuro Shirasaka, Akira Doshu-Kajiura, Mitsutaka Edanaga, Satoshi Tanaka, Saori Ikumi, Shingo Ito, Masayuki Okada, Tomoko Yorozu, for the SAPKA Investigators

**Affiliations:** 1 Department of Anesthesiology, Kyorin University School of Medicine, Tokyo, Japan; 2 Department of Anesthesia, Saitama Children’s Medical Center, Saitama, Japan; 3 Department of Anesthesiology, School of Medicine, International University of Health and Welfare, Chiba, Japan; 4 Department of Anesthesiology and Perioperative Medicine, Tohoku University Graduate School of Medicine, Miyagi, Japan; 5 Department of Hygiene and Public Health, Kyorin University School of Medicine, Tokyo, Japan; 6 Department of Diabetes, Endocrinology and Metabolism, Kyorin University School of Medicine, Tokyo, Japan; 7 Department of Anaesthesia and Intensive Care Medicine, Graduate School of Medicine and Faculty of Medicine, Akita University, Akita, Japan; 8 Department of Anesthesiology and Reanimatology, University of Fukui Hospital, Fukui, Japan; 9 Department of Anesthesiology, Fukuoka University School of Medicine, Fukuoka, Japan; 10 Department of Anesthesiology and Intensive Care, Hamamatsu University School of Medicine, Shizuoka, Japan; 11 Department of Anesthesiology, Hirosaki University Graduate School of Medicine, Hirosaki, Aomori Japan; 12 Department of Anesthesiology, The Jikei University School of Medicine, Tokyo, Japan; 13 Department of Anesthesiology and Intensive Care Medicine, Kawasaki Medical School, Okayama, Japan; 14 Department of Anesthesiology and Critical Care Medicine, Graduate School of Medical Sciences, Kyushu University, Fukuoka, Japan; 15 Department of Anesthesiology and Intensive Care, Faculty of Medicine, University of Miyazaki, Miyazaki, Japan; 16 Department of Anesthesiology, Nihon University School of Medicine, Tokyo, Japan; 17 Department of Anesthesiology, Sapporo Medical University School of Medicine, Hokkaido, Japan; 18 Department of Anesthesiology and Resuscitology, Shinshu University School of Medicine, Nagano, Japan; 19 Department of Anesthesiology, Tokyo Dental College Ichikawa General Hospital, Chiba, Japan; 20 Department of Anesthesiology, Faculty of Medicine, Yamagata University, Yamagata, Japan

**Keywords:** Sodium–glucose cotransporter 2 inhibitors, Diabetes mellitus, Diabetic ketoacidosis, Prospective study

## Abstract

**Purpose:**

Sodium-glucose cotransporter 2 inhibitors (SGLT2is) are commonly prescribed anti-diabetic medications with various beneficial effects; however, they have also been associated with ketoacidosis. The aim of this study was to determine the incidence of SGLT2i-associated perioperative ketoacidosis (SAPKA) in surgical patients.

**Methods:**

We conducted a multicenter, prospective cohort study across 16 centers in Japan, enrolling surgical patients with diabetes who were prescribed SGLT2is between January 2021 and August 2022. Patients were monitored until the third postoperative day to screen for SAPKA, defined as urine ketone positivity with a blood pH of < 7.30 and HCO_3_ level ≤ 18.0 mEq/L, excluding cases of respiratory acidosis.

**Results:**

In total, 759 of the 762 evaluated patients were included in the final analysis. Among these, three patients (0.40%) had urine ketones with a blood pH of < 7.30; however, blood gas analysis revealed respiratory acidosis in all three, and none of them was considered to have SAPKA. The estimated incidence of SGLT2i-associated postoperative ketoacidosis was 0% (95% confidence interval, 0%–0.4%).

**Conclusions:**

The observed incidence of SAPKA in our general surgical population was lower than expected. However, given that the study was observational in nature, interpretation of study results warrants careful considerations for biases.

**Supplementary Information:**

The online version contains supplementary material available at 10.1007/s00540-024-03335-3.

## Introduction

Sodium–glucose cotransporter 2 inhibitors (SGLT2is) represent a novel class of anti-diabetic medications that have been clinically shown to lower blood sugar levels, as well as induce weight loss, reduce blood pressure, and exert protective effects on the cardiovascular and renal systems [1–6]. Accordingly, SGLT2is have been increasingly prescribed owing to their various beneficial effects.

Despite their generally favorable safety profile and minimal adverse effects, SGLT2 is are also associated with ketoacidosis, with an incidence ranging from 1.4 to 8.8 events per 1,000 patient years among patients with diabetes who take these medications [7–12]. The risk of ketoacidosis may be higher in the perioperative setting, which is characterized by both fasting and surgical stress. Several studies indicate that these factors play a significant role in the onset of SGLT2i-associated perioperative ketoacidosis (SAPKA) [10–13]. Due to increased metabolic variability, reintroduction of oral nourishment, and augmented stress from surgery, individuals on SGLT2is require special attention during the postoperative period, as these factors can collectively elevate the risk of developing SAPKA. In 2020, the package inserts of SGLT2i were updated in some countries, advising a more extended preoperative cessation period—3 days for canagliflozin, dapagliflozin, and empagliflozin and 4 days for ertugliflozin, compared with the earlier recommendation of 24 h—to mitigate the risk of ketoacidosis. As the number of SAPKA case reports continues to grow, it is clear that merely retrospectively examining these cases is insufficient to gain a thorough understanding of this complication, which is crucial for devising appropriate preventive measures.

With the increasing use of SGLT2is for their beneficial pharmacologic effects and safety, a better understanding of the clinical epidemiology of SAPKA is crucial for the increasing number of patients undergoing surgery. Diagnosis of SAPKA is challenging, as many patients exhibit normal or only slightly elevated blood glucose levels, commonly referred to as euglycemic diabetic ketoacidosis. In addition, it presents with nonspecific symptoms such as nausea, vomiting, tachypnea, and abdominal pain, all of which are common among postoperative patients. These factors can lead to delayed diagnoses; therefore, prospective studies with rigorous diagnostic criteria are required to accurately identify SAPKA cases. This study aimed to ascertain the incidence of SAPKA in surgical patients undergoing general anesthesia who were prescribed SGLT2is for diabetes management.

## Methods

### Study design and patient selection

This multicenter, prospective cohort study was conducted across 16 university hospitals in Japan between January 2021 and August 2022. The rationale and details of the study protocol, including the statistical analysis plan, have been previously reported [14]. The eligibility criteria in our study were as follows: patients aged ≥ 20 years, diagnosed with diabetes and currently being treated with SGLT2is, and scheduled for surgery under general anesthesia. We excluded patients who were diagnosed with ketoacidosis at the time of screening or anesthesia induction, as well as those who had discontinued SGLT2is more than 1 week prior to surgery. In addition, our study did not include patients who underwent surgeries without general anesthesia as the shorter required fasting time before their procedure likely lessened their chances of developing ketoacidosis.

The study protocol was approved by the relevant institutional review board or ethics committee of each institution and was conducted according to the tenets of the Declaration of Helsinki. The ethics approval numbers for each center are listed in Online Resource 2. All patients provided written informed consent. The study team was entirely responsible for the study design and data analysis and collection, and the authors vouch for the accuracy and completeness of the data. The results reported here followed the Strengthening the Reporting of Observational Studies in Epidemiology guidelines for cohort studies [15].

### Study procedures

Perioperative management, including the preoperative cessation of SGLT2is and subsequent control of perioperative blood glucose, adhered to the routine practices of the participating institutions. Each institution applied its protocols, which were developed based on current clinical guidelines and tailored to meet the needs of their patient population.

Clinical and laboratory data were obtained at enrollment (baseline) by a trained investigator. Urine samples were collected on postoperative days (PODs) 0, 1, 2, and 3. If the patient was discharged from the hospital within 3 days after surgery, urine samples were collected until the day of discharge. If urine ketone positivity was detected, arterial or venous blood was collected to measure the blood pH; bicarbonate (HCO_3_) concentration; glucose concentration; and concentration of electrolytes, including sodium, potassium, and chloride. In patients in whom blood gas analysis (BGA) was performed as a routine postoperative examination, its results were also checked. After POD 3, the investigator at each site determined whether the patient developed ketoacidosis by checking the patient’s medical records until discharge. Data were collected using a paper-based case report form that was designed specifically for this study; after auditing the data quality, the forms were uploaded on the UMIN Internet Data and Information system for Clinical and Epidemiological research by a data manager at each site. A complete list of variables collected in this study can be found in Online Resource 3.

### Outcome measures

The primary outcome measure was the incidence of SAPKA within the first 3 days after surgery. In the published study protocol [14] and as supported by several guidelines [16–20], SAPKA was defined as the presence of urine ketones combined with a blood pH < 7.3 before beginning patient recruitment; however, during our study, we encountered instances where respiratory acidosis coincided with positive urine ketone results. Such findings raised concerns about potential misclassifications. Thus, to clearly distinguish SAPKA from respiratory acidosis and to ensure a more accurate diagnosis, we refined our criteria. In addition to the abovementioned urine ketone and pH criteria, we incorporated HCO_3_ values ≤ 18 mEq/L and an increased anion gap into our diagnostic framework.

### Statistical analyses

We hypothesized that the occurrence of SAPKA among patients on preoperative SGLT2i maintenance treatment would be > 0.5% based on previous research using insurance claims and adverse event data [7–12]. Using a Poisson distribution, we calculated that we would require at least 600 patients to confirm this hypothesis with 95% confidence [21]. We anticipated a 20% missing data rate due to potential difficulties in obtaining complete information for all patients during the study; therefore, we aimed to enroll a total of 750 patients to account for this expected data loss.

**Fig. 1:**
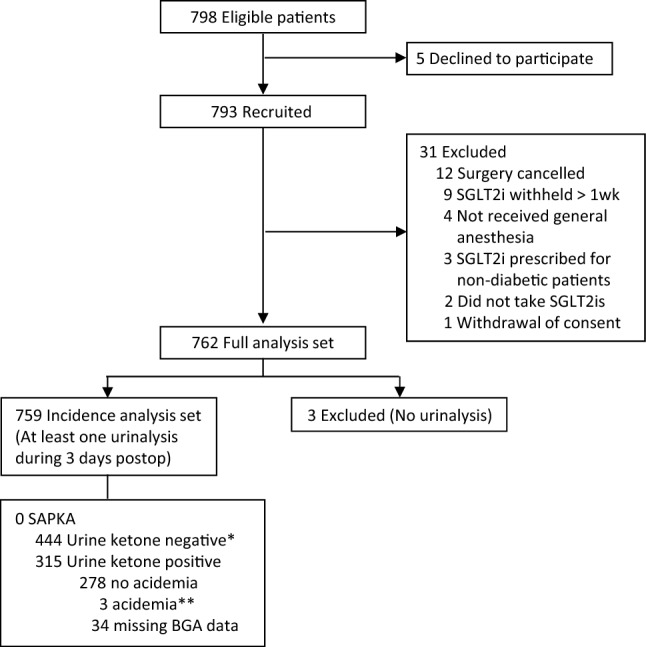
Study enrollment. *Among the 444 patients with negative urine ketone results, BGA was performed for 152 patients as part of routine postoperative examinations. Within this group, six instances of acidemia were identified: four of respiratory acidosis and two of metabolic acidosis. Notably, the two metabolic acidosis cases exhibited a normal anion gap. **Patients exhibited respiratory acidosis, evidenced by an elevated partial pressure of carbon dioxide and normal bicarbonate and anion gap values; no signs of ketoacidosis were detected. BGA, blood gas analysis; SAPKA, SGLT2i-associated postoperative ketoacidosis; SGLT2i, sodium–glucose cotransporter-2 inhibitor

Baseline characteristics and perioperative variables were recorded and analyzed using descriptive statistics. Continuous variables are expressed as the mean ± standard deviation or median with interquartile range, depending on the normality of the data distribution. Categorical variables are presented as frequencies and percentages. The incidence of SAPKA was calculated as the ratio of patients meeting the diagnostic criteria to the total number of patients with postoperative urinalysis data (incidence analysis set, Fig. 1). The 95% confidence interval for this incidence was calculated using a conventional method for proportions, considering both the sample size and proportion of patients diagnosed with SAPKA. If > 10% of the recruited patients had missing postoperative urinalysis data, multiple imputation by chained equations was performed to fill in the missing information [22]. All statistical analyses were performed using R version 3.6.3 (The R Foundation, Vienna, Austria), and SPSS software (IBM Corporation, Armonk, NY, USA).

## Results

### Baseline patient characteristics

The patient enrollment process is depicted in Fig. 1. Among the 798 patients initially screened, 5 declined to participate in the study, and 31 were excluded, as detailed in Fig. 1; thus, the full cohort for analysis comprised a total of 762 patients. Among the patients in the cohort, 3 were excluded owing to the unavailability of their urinalysis data, resulting in 759 patients being included in the primary outcome analysis for the incidence of SAPKA.

The baseline characteristics of these patients are shown in Table 1. Overall, 473 (62.3%) patients had an American Society of Anesthesiologists Physical Status classification of 2, and only 2 were emergency patients. The SGLT2is of all 759 patients were withheld until the day of surgery; 35.4% (269 patients) had a withholding period of > 3 days, while the rest were subject to a preoperative withholding period of ≤ 3 days. At the time of the preoperative screening, 153 (20.2%) patients were receiving insulin. Among 645 patients who underwent urinalysis at the time of preoperative screening, 29 (4.5%) had detectable urine ketones; despite having detectable urine ketones, these patients did not exhibit any additional clinical indications for SAPKA via BGA. Consequently, as there was no further cause to explore ketoacidosis, BGAs were not performed preoperatively in any patient.

**Table 1:** Baseline characteristics of the patients (*n* = 759)

Median age, years (IQR)	68	(59, 74)
Male sex, no. (%)	505	(66.5)
Median height, cm (IQR)	164.0	(155.6, 170.0)
Median body weight, kg (IQR)	67.0	(59.7, 77.4)
Median body mass index^a^, kg m^−2^ (IQR)	25.3	(22.7, 28.4)
ASA physical status classification, no. (%)^b^		
1	0	(0.0)
2	473	(62.3)
3	278	(36.6)
4	6	(0.9)
1E	0	(0.0)
2E	0	(0.0)
3E	2	(0.3)
4E	0	(0.0)
Smoking status, no. (%)		
Never	297	(39.1)
Past	370	(48.7)
Current	92	(12.1)
Diabetes mellitus, no. (%)		
Type 1	13	(1.7)
Type 2	746	(98.3)
Duration since diagnosis, no. total no.^−1^ (%)		
< 1 year	25/759	(3.3)
≤ 1 year, < 5 years	102/759	(13.4)
≤ 5 years, < 10 years	109/759	(14.4)
≥ 10 years	269/759	(35.4)
Unknown	254/759	(33.5)
Glucose-lowering therapies, no. (%)		
SGLT2is		
Canagliflozin, no. (%)	75	(9.9)
Canagliflozin + teneligliptin^c^, no. (%)	44	(5.8)
Dapagliflozin, no. (%)	185	(24.4)
Empagliflozin, no. (%)	183	(24.1)
Empagliflozin + linagliptin^c^, no. (%)	42	(5.5)
Ipragliflozin, no. (%)	92	(12.1)
Ipragliflozin + sitagliptin^c^, no. (%)	38	(5.0)
Luseogliflozin, no. (%)	53	(7.0)
Tofogliflozin, no. (%)	47	(6.2)
Other agents, no. (%)		
Insulin	153	(20.2)
Alpha glucosidase inhibitors	40	(5.3)
Biguanide	252	(33.2)
DPP4is	272	(35.8)
Glinides	36	(4.7)
GLP-1 receptor agonists	1	(0.1)
Sulphonyl urea	77	(10.1)
Thiazolidines	23	(3.0)
DPP4i + biguanide	48	(6.3)
DPP4i + thiazolidine	3	(0.4)
Glinides + alpha glucosidase inhibitors	12	(1.6)
Thiazolidine + biguanide	3	(0.4)
Thiazolidine + sulfonyl urea	0	(0.0)
Duration since SGLT2i initiation, no. total^−1^		
< 6 M	70/759	(9.2)
≥ 6 M, < 1 year	30/759	(4.0)
≥ 1 year, < 2 years	54/759	(7.1)
> 2 years	122/759	(16.1)
Unknown	483/759	(63.6)
Comorbidities, no. (%)		
Hypertension	578	(76.2)
Dyslipidemia	484	(63.8)
Hyperuricemia	112	(14.8)
Liver dysfunction	99	(13.0)
Renal dysfunction	268	(35.3)
Chronic obstructive pulmonary diseases	56	(7.4)
Hemodialysis	1	(0.1)
Coronary artery disease	186	(24.5)
Heart failure	74	(9.7)
Stroke	67	(8.8)
Atherosclerosis obliterans	38	(5.0)
Carotid artery stenosis	38	(5.0)
Medications, no. (%)		
Antihypertensive drugs	555	(73.1)
Diuretics	121	(15.9)
Antiplatelet agents	197	(26.0)
Anticoagulants	86	(11.3)
Bronchodilators	38	(5.0)
Preoperative laboratory data, median (IQR)		
Glycated hemoglobin level, %	6.9	(6.5, 7.4)
Glucose level, mg/dL	130	(109, 159)
Glomerular filtration rate, mL/min	65.9	(51.2, 82.0)
Serum creatinine level, mg/dL	0.83	(0.67, 1.0)
Hemoglobin level, g/dL	14.2	(12.7, 15.4)
Hematocrit, %	43.0	(39.1, 46.4)
Aspartate aminotransferase level, IU/L	21.0	(17.0, 27.0)
Alanine aminotransferase level, IU/L	20.0	(14.0, 29.0)
Gamma-glutamyl transferase level, IU/L	26.0	(18.0, 45.0)
Sodium level, mmol/L	140.0	(139.0, 142.0)
Potassium level, mmol/L	4.2	(4.0, 4.5)
Chloride level, mmol/L	104.0	(102.0, 106.0)
Preoperative urinalysis no./total, no. (%)		
Urine ketone, ≥ 1 +	29/759	(3.8)
Urine sugar		
Negative	47/759	(6.2)
1 +	8/759	(1.1)
2 +	14/759	(1.8)
3 +	79/759	(10.4)
4 +	497/759	(65.5)
Not measured	114/759	(15.0)
Cessation of SGLT2is, no./total no. (%)		
0 d (last dose taken on the day of surgery)	2/759	(0.3)
1 d (last dose taken on 1 day before surgery)	260/759	(34.3)
2 d (last dose taken on 2 days before surgery)	106/759	(14.0)
3 d (last dose taken on 3 days before surgery)	122/759	(16.1)
> 3 d	269/759	(35.4)
Duration of preoperative fasting, no./total no. (%)		
< 4 h	6/759	(0.8)
4–12 h	191/759	(25.2)
12–24 h	534/759	(70.4)
24–72 h	26/759	(3.4)
≥ 72 h	2/759	(0.3)
Preoperative insulin administration on the day of surgery, no./total no. (%)	99/758	(13.1)
Intraoperative insulin administration, no. total no. ^−1^ (%)	48/758	(6.3)
Intraoperative glucose administration, no. (%)	702/759	(92.5)
Surgical site, no. (%)		
Abdominal		
Laparoscopic	162	(21.3)
Laparotomy	92	(12.1)
Other	3	(0.4)
Breast	21	(2.8)
Cardiovascular		
CABG	23	(3.0)
Other cardiac	38	(5.0)
Aortic	6	(0.8)
Peripheral artery	9	(1.2)
Endovascular	10	(1.3)
Chest wall, abdominal wall, perineal	23	(3.0)
Cesarean section	0	(0.0)
Eye, ear, nose, throat, head	104	(13.7)
Neuro	29	(3.8)
Orthopedic		
Spine	61	(8.0)
Extremities	87	(11.5)
Thoracic	35	(4.6)
Thoracoabdominal	5	(0.7)
Trans-urethral or -vaginal	32	(4.2)
Other	19	(2.5)
Type of anesthesia, no. (%)		
General anesthesia alone	497	(65.5)
General + regional anesthesia	262	(34.5)
Type of general anesthesia, no. (%)		
Volatile	543	(71.5)
Intravenous	216	(28.5)
Median duration of surgery, min (IQR)	168	(102, 285)
Median duration of anesthesia, min (IQR)	243	(166, 370)
Intensive care unit admission, no. (%)	187	(24.6)
Postoperative mechanical ventilation, no. (%)	57	(7.5)

^a^The body mass index is the weight in kilograms divided by the square of the height in meters

^b^American Society of Anesthesiologists (ASA) physical status classes range from 1 to 5, with higher classes indicating more severe systemic disease. “E” indicates emergency surgery

^c^Combination tablet

Percentages may not total 100 due to rounding

*CABG* coronary artery bypass grafting, *DPP4* dipeptidyl peptidase-4, *GLP-1* glucagon-like peptide-1, *IQR* interquartile range, *M* month; *SGLT2i* sodium–glucose cotransporter 2 inhibitor

Our study included a diverse range of surgical procedures, including abdominal, breast, cardiovascular, chest wall/abdominal wall/perineal, eye/ear/nose/throat/head, neurological, orthopedic, thoracic, thoracoabdominal, and trans-urethral or -vaginal surgeries, reflecting a wide spectrum of surgical disciplines. All patients received general anesthesia with or without regional anesthesia, and 92.5% of the study population received intraoperative glucose-containing intravenous fluids. Intraoperative insulin was administered in 6.3% of the patients. In addition, no intraoperative critical adverse events were reported.

After the surgery, 24.6% of the patients were admitted to the intensive care unit, where 7.5% required mechanical ventilation (Table 1). By POD 3, insulin had been administered at least once to 444 (58.5%; Online Resource 4) patients. Furthermore, 360 (47.4%) patients resumed taking SGLT2is within 3 days after surgery. Notably, no in-hospital mortality was observed.

### Outcome measures

During the initial 3 days after surgery, a total of 2,943 urinary samples were collected from the 759 study participants and analyzed for urine ketones. By POD 3, 315 (41.5%) patients tested positive for urine ketones at least once; among them, BGA was conducted on 281 (89.2%) patients. Only three patients exhibited a pH value lower than the threshold for SAPKA (pH < 7.30)—two on the day of surgery and one on POD 1. On further analysis, the BGA for these three individuals indicated respiratory acidosis, with elevated partial pressure of carbon dioxide and HCO_3_ and anion gap values within the normal range, rather than diabetic ketoacidosis (Table 2). Considering our refined diagnostic criteria, no SAPKA cases were identified in this study. Thus, the estimated incidence rate of SAPKA remained at 0%, with a 95% confidence interval in the range of 0.0–0.4%.

**Table 2:** Details of cases involving patients that developed postoperative acidosis

Res ID	Age	Sex	Surgery	Duration of anesthesia (min)	SGLT2is	Urine ketone	Day of examination (POD)	pH	HCO_3_ (mg/L)	PaCO_2_ (Torr)	AG (mg/L)	BG (mg/dL)
182	66	M	CABG	564	Empagliflozin	2 +	1	7.276	22.0	48.8	6.0	148
284	71	F	Orthopedic (spine)	339	Ipragliflozin	3 +	1	7.271	22.5	50.5	10.5	126
735	77	M	Orthopedic (spine)	114	Canagliflozin	2 +	0	7.292	22.5	53.5	10.5	144

*AG* anion gap, *BG* blood glucose, *CABG* coronary artery bypass grafting, *POD* postoperative day, *SGLT2i* sodium–glucose cotransporter 2 inhibitor

## Discussion

Despite the advantages of SGLT2 is as anti-diabetic medications, they have been linked to ketoacidosis. The incidence of SAPKA, a potentially critical condition, among surgical patients receiving ongoing SGLT2i therapy has been uncertain. This extensive cohort study reveals no occurrence of SAPKA, implying that its incidence might be less than anticipated. Previous epidemiologic studies have focused on the incidence of diabetic ketoacidosis in patients prescribed SGLT2is, reporting an incidence rate of 1.4–8.8 events per 1,000 patient years. A recent population-based cohort study targeting surgical patients estimated an incidence of 6.4 events per 1,000 patient years [23]. In contrast to our current study’s prospective design, the population-based study design previously employed may be limited by potential confounding factors and biases, impacting its generalizability and applicability to broader populations. Although case reports of SAPKA have been rapidly increasing, they provide limited information on the robust epidemiology of SAPKA. Our study thus offers valuable insights into the incidence and management of SAPKA in the perioperative setting, with refined criteria for detection.

The absence of SAPKA cases in our study population can be attributed to several factors; first, selection bias cannot be ruled out as a possible explanation, as patients with a lower risk of SAPKA may have been disproportionately included. A primary exclusion in our study was the removal of patients diagnosed with ketoacidosis at the time of screening or induction of anesthesia. The exclusion of this group, perhaps the most at-risk cohort for SAPKA, may have led to underestimating the condition’s incidence. Our study, which was initiated in 2021, considered the evolving guidelines on preoperative SGLT2i discontinuation. While Japanese guidelines suggest a 24-h withdrawal, the Diabetes Society recommends a 3-day period. This variance resulted in diverse practices at our study sites. It is crucial to understand that our study’s purpose was not to confirm a specific drug withdrawal timeframe but rather to examine the incidence of SAPKA. Our findings do not advocate for or against specific withdrawal periods. Despite mixed adherence to withdrawal guidelines, no SAPKA cases were observed, even in the 35.4% of patients who discontinued SGLT2is for < 3 days, which we found noteworthy. This aligns with literature indicating that an extended withdrawal period may reduce the risk of developing SAPKA [24]. The scarcity of emergency operations in our study population is another possible factor that could explain the lack of SAPKA occurrences. Emergency surgery has been identified as a potential risk factor for SAPKA, and accounted for 24% of reported cases in a previous systematic review [24]. Therefore, a lower number of high-risk patients could have resulted in underestimation of the incidence of SAPKA. Although our study protocol intended to include emergency surgery cases, the urgency associated with such procedures often precluded the comprehensive process of obtaining informed consent for enrollment, potentially leading to their underrepresentation. In emergency cases, it may be challenging to implement appropriate preoperative withholding periods for SGLT2is; therefore, owing to these possible selection biases, the external validity of our findings regarding other surgical populations may be limited.

Second, our study underscores the challenge of information bias resulting from the evolving diagnostic criteria for SAPKA. Diagnosing SAPKA remains intricate owing to its nonspecific symptoms and euglycemic ketoacidosis. Although elevated serum ketone levels are a defining feature of SAPKA, point-of-care capillary ketone measurement is not universally accessible. In our study, BGA was performed for 152 patients without urine ketones, revealing acidosis in six cases; still, none presented with ketoacidosis (Online Resource 5). Among the 34 patients with urine ketones without BGA data, their clinical trajectories did not suggest the presence of SAPKA (Online Resource 6). Even with the testing constraints, we maintain confidence in the low risk of overlooking SAPKA cases within our cohort. This assurance is grounded in the meticulous clinical evaluations and ongoing monitoring given to patients, regardless of specific laboratory data availability. Such an intensive approach allowed for the identification of potential SAPKA indicators. In response to these diagnostic challenges, we have refined the definition of SAPKA to encompass urine ketone positivity, a blood pH < 7.3, an HCO_3_ level ≤ 18 mEq/L, and an increase in the anion gap. This adjustment is crucial, as respiratory acidosis is prevalent among postoperative patients and acidosis of respiratory origin might have been mistakenly linked to SAPKA when relying solely on urinary ketone and pH criteria. The urgent need for comprehensive and standardized diagnostic criteria for SAPKA in the perioperative context calls for further investigative efforts.

Third, several confounders must be considered to understand our study’s absence of SAPKA events. In our cohort, 92.5% of the patients received intraoperative glucose administration, and 58.5% received postoperative insulin at least once by POD 3 (Table 1 and Online Resource 4). Infusion of small doses of glucose during fasting has been shown to reduce the risk of ketosis, as insulin deficiency and carbohydrate deprivation play pivotal roles in the pathophysiology of SAPKA [25, 26]. It is unclear whether intraoperative glucose supplementation was administered as a routine management approach or whether practitioners intentionally used glucose-containing solutions to prevent ketosis. In Japan, 1% glucose-containing Ringer’s solution is commonly used as an intraoperative fluid. Intraoperative glucose supplementation and postoperative insulin control in a significantly higher percentage of the study patients may have acted as confounders, potentially leading to a lower incidence of SAPKA.

When addressing sample size and statistical considerations in our observational cohort study, we acknowledge the challenges associated with estimating the incidence of rare events such as SAPKA. In our study, we predicted an incidence of SAPKA greater than 0.5%, and appropriately powered the study with a sample size of 762 patients. This sample size, surpassing our initial projections, was selected to enhance the precision of our incidence estimation within reasonable cost constraints. The absence of observed SAPKA events suggests a potentially low incidence, reflecting the effectiveness of current perioperative management. However, it also demonstrates the limitations of cohort studies in assessing rare outcomes. Considering the exponential increase in resource requirements and diminishing returns associated with increased sample size, future research may benefit from alternative methodologies. Population-based studies leveraging large healthcare databases offer a more pragmatic approach, allowing for broader generalizability and efficient utilization of resources while addressing rare clinical events such as SAPKA [23].

Our study has several important strengths that enhance the validity and impact of the results. First, the multicenter design of the study increases its generalizability, making the findings more applicable to a wider population. Second, the large sample size enhances the precision and accuracy of our estimates, providing more robust conclusions. Third, defining the population of surgical patients using SGLT2is helped to increase the study’s internal validity and minimize the risk of confounding. Fourth, the prospective design reduces the risk of bias and improves the reliability of the results. Finally, our study addresses an important and understudied topic in the literature, making a valuable contribution to the field. The study findings thus provide essential insights into the incidence of SAPKA in surgical patients with diabetes. However, our study also has some limitations. As discussed previously, selection, information, and confounding biases may have influenced our results, as the study population did not fully represent all surgical patients using SGLT2is. Consequently, the external validity of our study results should be considered with caution. Second, the absence of SAPKA cases may have limited our ability to understand the incidence of this condition fully; thus, more extensive studies may be required for comprehensive data on SAPKA incidence.

Nonetheless, our study offers valuable information for a comprehensive understanding of the epidemiology of SAPKA. As the use of SGLT2is continues to increase, future research should focus on identifying high-risk patient groups for SAPKA and developing effective perioperative management protocols to minimize the risk of this potentially life-threatening condition.

In conclusion, this study observed a lower incidence of SAPKA in the general surgical population than anticipated, suggesting that SGLT2i administration under current standard practices does not significantly increase the risk of perioperative ketoacidosis. However, given the observational nature of this study, it is important to interpret these results with caution and acknowledge potential biases. These findings underscore the need for more stringent diagnostic criteria and standardized screening protocols for SAPKA in a perioperative setting, which would help to enhance diabetes management in surgical patients.

### Supplementary Information

Below is the link to the electronic supplementary material.

Supplementary file1 (DOCX 47 KB)

## Data Availability

The data supporting this study’s findings are available from the corresponding author upon reasonable request.
