# Supplementary material for: Incidence of sodium–glucose cotransporter-2 inhibitor-associated perioperative ketoacidosis in surgical patients: a prospective cohort study
Source: J Anesth. 2024 Mar 17;38(4):464–74. doi: 10.1007/s00540-024-03335-3 (PMC11284185; doi:10.1007/s00540-024-03335-3)
Supplement: Supplementary file 1 — Supplementary file1 (DOCX 47 KB) [file 540_2024_3335_MOESM1_ESM.docx]

**Incidence of Sodium-Glucose Cotransporter-2 Inhibitor-Associated Perioperative Ketoacidosis in Surgical Patients: A Prospective Cohort Study**

**Supplementary Data**

*Hiroyuki Seki, MD, PhD, Norifumi Kuratani, MD, PhD, MPH, Toshiya Shiga, MD, PhD, Yudai Iwasaki, MD, Kanae Karita, MD, PhD, MHS, Kazuki Yasuda, MD, PhD, Natsuko Yamamoto, MD, Yuko Nakanishi, MD, Kenji Shigematsu, MD, Kensuke Kobayashi, MD, PhD, Junichi Saito, MD, Ichiro Kondo, MD, PhD, Nozomi Yaida, MD, Hidenobu Watanabe, MD, Midoriko Higashi, MD, PhD, Tetsuro Shirasaka, MD, PhD, Akira Doshu-Kajiura, MD, PhD, Mitsutaka Edanaga, MD, PhD, Satoshi Tanaka, MD, Saori Ikumi, MD, Shingo Ito, MD, Masayuki Okada, MD, Tomoko Yorozu, MD, PhD, for the SAPKA Investigators

*Corresponding author: Hiroyuki Seki, MD, PhD

Department of Anesthesiology, Kyorin University School of Medicine

Mailing address: 6-20-2 Shinkawa Mitaka, Tokyo 181-8611, Japan

Tel: 81-422-47-5511

E-mail: hseki@ks.kyorin-u.ac.jp

Table of contents

Online Resource 1. List of SAPKA investigators

Online Resource 2. List of the approval numbers for each center

Online Resource 3. List of variables collected in the SAPKA study

Online Resource 4. Postoperative variables

Online Resource 5. Details of cases developed postoperative acidosis in whom urine ketone was negative, but BGA was performed as a routine postoperative examination

Online Resource 6. Details of cases with urine ketone positive but missing blood gas analysis

**Online Resource 1. SAPKA investigators**

| **Akita University** | **ORCHiD** |
| --- | --- |
| Yukitoshi Niiyama, MD, PhD | 0009-0002-4216-7995 |
|  |  |
| **Fukui University** |  |
| Shuko Matsuda, MD | 0000-0002-1064-0015 |
| Yuka Matsuki, MD, PhD | 0000-0003-2949-2574 |
| Kenji Shigemi, MD, PhD | 0000-0002-3281-4477 |
| Yukiko Suzuki, MD | 0000-0001-8786-4722 |
|  |  |
| **Fukuoka University** |  |
| Shozo Tominaga, MD | 0000-0001-7104-5036 |
|  |  |
| **Hamamatsu University** |  |
| Yoshitaka Aoki, MD | 0000-0002-3750-4160 |
| Hitomi Asaba, MD | 0000-0003-3411-8149 |
| Ryo Imai, MD | 0000-0002-3142-3685 |
| Takayuki Katsuragawa, MD | 0000-0002-3339-0629 |
| Shingo Kawashima, MD, PhD | 0000-0001-5474-2534 |
| Soichiro Mimuro, MD, PhD | 0000-0002-3854-9950 |
| Yoshiki Nakajima, MD, PhD | 0000-0001-6831-8515 |
| Satoshi Naruse, MD | 0000-0002-9925-2747 |
| Sho Sugimura, MD | 0000-0002-3401-5900 |
| Kota Suzuki, MD | 0000-0003-3586-1102 |
| Yuji Suzuki, MD | 0000-0003-1156-3311 |
|  |  |
| **Hirosaki University** |  |
| Naoki Hirai, MD | 0000-0002-1364-3326 |
| Kazuyoshi Hirota, MD, FRCA | 0000-0001-6936-0985 |
| Hirotaka Kinoshita, MD | 0000-0003-4490-3941 |
| Mirei Kubota, MD | 0000-0002-8396-0386 |
| Daiki Takekawa, MD | 0000-0003-4005-9536 |
| Hiromasa Tsuyama | 0000-0003-1543-5667 |
|  |  |
| **The Jikei University** |  |
| Kohei Ikeda, MD | 0000-0002-0961-1532 |
| Yoshihiro Kimura, MD | 0000-0003-0817-3224 |
| Masayoshi Koike, MD | 0000-0002-2741-9210 |
| Koji Takano, MD | 0000-0002-0561-8542 |
| Kazunari Takahashi, MD | 0000-0003-3836-5091 |
| Yu kawada, MD | 0000-0001-8934-4333 |
|  |  |
| **Kawasaki Medical School** |  |
| Myoichiro Maeshima, MD, PhD | 0000-0003-1057-0173 |
|  |  |
| **Kyorin University** |  |
| Chihiro Akisawa, MD | 0000-0002-5622-5757 |
| Shogo Ema, MD | 0000-0002-8512-5659 |
| Mugi Kawafune, MD | 0000-0003-0619-5585 |
| Eri Komatsu, MD | 0000-0002-9040-9277 |
| Kazuya Kondo, MD | 0000-0002-5101-5128 |
| Misa Koshihara, MD | 0000-0001-8394-0863 |
| Hironori Motoyama, MD | 0000-0002-7229-6803 |
| Hikari Noguchi, MD | 0000-0003-3102-4999 |
| Motoaki Ono, MD | 0000-0002-0593-0026 |
| Kaio Okamura, MD | 0000-0001-7279-3562 |
| Yukiko Saito, MD | 0000-0002-2161-7478 |
| Keisuke Shimazu, MD | 0000-0003-0092-4599 |
| Takahiro Yoshikawa, MD | 0000-0002-0434-5271 |
|  |  |
| **Kyushu University** |  |
| Tetsuhiro Fujiyoshi, MD, PhD | 0000-0002-6788-7633 |
| Aiko Maeda, MD, PhD | 0000-0003-2496-6865 |
| Kazuhiro Shirozu, MD, PhD | 0000-0003-2290-3764 |
| Shoko Nakayama, MD | 0000-0002-2285-8441 |
| Makoto Sumie, MD, PhD | 0000-0003-1001-8872 |
| Katsuyuki Matsushita, MD, PhD | 0000-0002-0466-0861 |
| Misaki Yamamoto, MD, PhD | 0000-0003-4689-3054 |
| Taku Nakagawa, MD, PhD | 0000-0002-1552-1752 |
| Keiko Nobukuni, MD | 0000-0001-9730-5406 |
| Yukie Matsubara, MD, PhD | 0000-0003-4107-4156 |
| Shoichi Sasaki, MD | 0000-0002-1355-2555 |
|  |  |
| **Sapporo Medical University** |  |
| Kengo Hayamizu, MD, PhD | 0000-0003-4095-5189 |
| Satoshi Sato, MD | 0000-0002-4487-8515 |
| Sayaka Sotome, MD | 0000-0001-7916-6270 |
| Atsushi Kokita, MD | 0000-0003-3981-8689 |
| Hironori Kintaka, MD | 0000-0002-4126-5043 |
| Risa Yamaguchi, MD | 0000-0002-6459-3330 |
| Yuiko Mori, MD | 0000-0003-4761-9647 |
| Yukiko Takahashi, MD | 0000-0002-1617-9135 |
|  |  |
| **Shinshu University** |  |
| Masatoshi Urasawa, MD | 0000-0003-1664-8904 |
|  |  |
| **Tohoku University** |  |
| Daisuke Konno, MD, PhD | 0000-0001-9978-3647 |
| Hirona Nishimaki, MD | 0000-0001-6034-4129 |
| Daisuke Irimada, MD | 0000-0002-3119-7163 |
| Takahiro Tasaki, MD | 0000-0002-4326-5725 |
| Keisuke Yamashita, MD | 0000-0003-4928-350X |
|  |  |
| **Tokyo Dental College Ichikawa General Hospital** |  |
| Yoshihiko Deguchi, MD | 0000-0003-2789-3833 |
| Yasuhi Innami, MD, PhD | 0000-0002-7635-3588 |
| Hiroyuki Inoue, DDS | 0000-0002-9847-1550 |
| Tomofumi Kodaira, MD | 0000-0002-3790-5722 |
| Reina Okada, DDS | 0000-0002-1184-5696 |
| Takashi Ouchi, MD, PhD | 0000-0001-7022-6820 |

**Online Resource 2. List of the approval numbers for each center**

| Center | Approval number | Date of approval |
| --- | --- | --- |
| Akita university | 2686 | October 14, 2021 |
| Fukui university | 20200183 | August 23,, 2021 |
| Fukuoka university | H21-01-004 | February 8, 2021 |
| Hamamatsu university | 20-292 | January 6, 2021 |
| Hirosaki university | 2020-280 | January 29, 2021 |
| The Jikei university | 33-181 | September 13, 2021 |
| Kawasaki medical university | 5273-00 | June 8, 2021 |
| Kyorin university | 785 | October 26, 2020 |
| Kyushu university | 2020-699 | February 17, 2021 |
| University of Miyazaki | O-0895 | February 17, 2021 |
| Nihon university | RK-201208-3 | December 9, 2020 |
| Sapporo medical university | 322-226 | January 14, 2021 |
| Shinshu university | 5041 | February 16, 2021 |
| Tohoku university | 2021-1-490-1 | December 23, 2020 |
| Tokyo dental college Ichikawa general hospital | I 20-76 | February 9, 2021 |
| Yamagata university | 2020-369 | February 22, 2021 |

**Online Resource 3. List of variables collected in the SAPKA study**

| Preoperative values | Patients’ background | Age, yr |
| --- | --- | --- |
|  |  | Sex, male or female |
|  |  | Height, cm |
|  |  | Body weight, kg |
|  |  | Body-mass index, kg/m^2^ |
|  |  | ASA physical status,  I, II, III, IV, V, IE, IIE, IIIE, IVE, VE |
|  |  | Smoking status,  never, current, past |
|  | Diabetes mellitus | Type 1 or 2 |
|  |  | Duration since diagnosis,  < 1 yr  1 yr ≤, < 5 yr  5 yr ≤, < 10 yr  ≥ 10 yr  Unknown |
|  | Glucose-lowering therapies | Type of agents  SGLT2is  Canagliflozin  Canagliflozin + teneligliptin  Dapagliflozin  Empagliflozin  Empagliflozin + linagliptin  Ipragliflozin  Ipragliflozin + sitagliptin  Luseogliflozin  Tofogliflozin  Other agents  Insulin  Alpha-glucosidase inhibitors  Biguanide  DPP4is  Glinides  GLP-1 receptor agonist  Sulphonyl urea  Thiazolidines  DPP4i + biguanide  DPP4i + thiazolidine  Glinides + alpha glucosidase inhibitors  Thiazolidine + biguanide  Thiazolidine + sulfonyl urea |
|  | SGLT2is | Duration since initiation  < 6 M  6M ≤, < 1 yr  1 yr ≤, < 2 yr  2 yr <  Unknown |
|  | Comorbidities | Hypertension |
|  |  | Dyslipidemia |
|  |  | Hyperuricemia |
|  |  | Liver dysfunction |
|  |  | Renal dysfunction |
|  |  | Chronic obstructive pulmonary diseases |
|  |  | Hemodialysis |
|  |  | Coronary artery disease |
|  |  | Heart failure |
|  |  | Stroke |
|  |  | Atherosclerosis obliterans |
|  |  | Carotid artery stenosis |
|  | Medications | Antihypertensive |
|  |  | Diuretics |
|  |  | Antiplatelets |
|  |  | Anticoagulants |
|  |  | Bronchodilators |
|  | Preoperative laboratory data at the time of preoperative screening | Glycated hemoglobin level, % |
|  |  | Glucose level, mg/dl |
|  |  | Glomerular filtration rate, ml/min |
|  |  | Serum creatinine, mg/dl |
|  |  | Hemoglobin, g/dl |
|  |  | Hematocrit, % |
|  |  | Aspartate aminotransferase, IU/L |
|  |  | Alanine aminotransferase, IU/L |
|  |  | Gamma-glutamyl transferase, IU/L |
|  |  | Sodium, mmol/L |
|  |  | Potassium, mmol/L |
|  |  | Chloride, mmol/L |
|  | Preoperative urinalysis at the time of preoperative screening | Urine ketone  ≥ 1+ |
|  |  | Urine sugar  Negative  1+  2+  3+  4+  Not measured |
|  | Cessation of SGLT2is | 0d (last dose taken on the day of surgery)  1d (last dose taken on 1 day before surgery)  2d (last dose taken on 2 days before surgery)  3d (last dose taken on 3 days before surgery)  More than 3d |
|  | Duration of preoperative fasting | < 4h  4 to 12h  12 to 24h  24 to 72h  ≥ 72h |
|  | Preoperative insulin on the day of surgery | Yes or no |
| Surgery | Surgical site | Abdominal |
|  |  | Laparoscopic |
|  |  | Laparotomy |
|  |  | Other |
|  |  | Breast |
|  |  | Cardiovascular |
|  |  | CABG |
|  |  | Other cardiac |
|  |  | Aortic |
|  |  | Peripheral artery |
|  |  | Endovascular |
|  |  | Chest wall, abdominal wall, perineal |
|  |  | Cesarean section |
|  |  | Eye, ear, nose, throat, head |
|  |  | Neuro |
|  |  | Orthopedic |
|  |  | Spine |
|  |  | Extremities |
|  |  | Thoracic |
|  |  | Thoracoabdominal |
|  |  | Trans-urethral or -vaginal |
|  |  | Other |
|  | Duration of surgery, min |  |
| Anesthesia | Type of anesthesia | General |
|  |  | General + regional |
|  | Type of general anesthesia | Volatile |
|  |  | Intravenous |
|  | Duration of anesthesia, min |  |
| Intraoperative variables | Intraoperative insulin administration | Yes or no |
|  | Intraoperative glucose administration | Yes or no |
| Postoperative variables | Intensive care unit admission | Yes or no |
|  | Postoperative mechanical ventilation | Yes or no |
|  | Symptoms, PODs 0-3, yes or no | Nausea |
|  |  | Vomiting |
|  |  | Abdominal pain |
|  |  | Tachypnea |
|  | Use of insulin | Yes or no |
|  | Postoperative urinalysis, PODs 0-3 | Urine ketone  ≥ 1+ |
|  |  | Urine sugar  Negative  1+  2+  3+  4+  Not measured |
|  | Date of oral intake resumption | Postoperative day 0 |
|  |  | Postoperative day 1 |
|  |  | Postoperative day 2 |
|  |  | Postoperative day 3 |
|  |  | Later than postoperative day 3 |
|  | SGLT2i restart | Postoperative day 0 |
|  |  | Postoperative day 1 |
|  |  | Postoperative day 2 |
|  |  | Postoperative day 3 |
|  |  | Later than postoperative day 3 |
|  |  | Not resumed |
|  | Ketoacidosis after postoperative day 3 | Yes or no |
|  | Duration of hospitalization after surgery, day |  |

CABG, coronary artery bypass grafting; DPP4, dipeptidyl peptidase-4; GLP-1, glucagon-like peptide-1; SGLT2, sodium-glucose cotransporter 2. *The body-mass index is the weight in kilograms divided by the square of the height in meters. ^†^American Society of Anesthesiologists (ASA) physical status classes range from 1 to 5, with higher classes indicating more severe systemic disease. “E” indicates emergency surgery.

**Online Resource 4. Postoperative variables**

| Nausea no./total no. (%) |  |  |
| --- | --- | --- |
| Postoperative day 0 | 85/727 | (11.7) |
| Postoperative day 1 | 51/754 | (6.8) |
| Postoperative day 2 | 21/742 | (2.8) |
| Postoperative day 3 | 9/722 | (1.2) |
| Vomiting no./total no. (%) |  |  |
| Postoperative day 0 | 34/727 | (4.7) |
| Postoperative day 1 | 17/754 | (2.3) |
| Postoperative day 2 | 6/741 | (0.8) |
| Postoperative day 3 | 2/722 | (0.3) |
| Abdominal pain no./total no. (%) |  |  |
| Postoperative day 0 | 96/725 | (13.2) |
| Postoperative day 1 | 95/751 | (12.6) |
| Postoperative day 2 | 73/739 | (9.9) |
| Postoperative day 3 | 54/720 | (7.5) |
| Tachypnea no./total no. (%) |  |  |
| Postoperative day 0 | 7/731 | (1.0) |
| Postoperative day 1 | 10/754 | (1.3) |
| Postoperative day 2 | 4/743 | (0.5) |
| Postoperative day 3 | 1/723 | (0.1) |
| Use of insulin no./total no. (%) |  |  |
| Postoperative day 0 | 262/759 | (34.5) |
| Postoperative day 1 | 382/759 | (50.3) |
| Postoperative day 2 | 339/744 | (45.6) |
| Postoperative day 3 | 297/722 | (41.1) |
| Urine ketone ≥1+ no./total no. (%) |  |  |
| Postoperative day 0 | 129/741 | (17.4) |
| Postoperative day 1 | 190/754 | (25.2) |
| Postoperative day 2 | 139/736 | (18.9) |
| Postoperative day 3 | 129/712 | (18.1) |
| Urine sugar no./total no. (%) |  |  |
| Postoperative day 0 |  |  |
| Negative | 277/741 | (37.4) |
| 1+ | 79/741 | (10.7) |
| 2+ | 81/741 | (11.0) |
| 3+ | 122/741 | (16.5) |
| 4+ | 180/741 | (24.4) |
| Postoperative day 1 |  |  |
| Negative | 199/754 | (26.4) |
| 1+ | 97/754 | (12.9) |
| 2+ | 105/754 | (13.9) |
| 3+ | 147/754 | (19.5) |
| 4+ | 206/754 | (27.3) |
| Postoperative day 2 |  |  |
| Negative | 250/735 | (34.0) |
| 1+ | 92/735 | (12.5) |
| 2+ | 82/735 | (11.2) |
| 3+ | 107/735 | (14.6) |
| 4+ | 204/735 | (27.8) |
| Postoperative day 3 |  |  |
| Negative | 227/711 | (31.9) |
| 1+ | 76/711 | (10.7) |
| 2+ | 79/711 | (11.1) |
| 3+ | 94/711 | (13.2) |
| 4+ | 235/711 | (33.1) |
| Date of oral intake resumption no. (%) |  |  |
| Postoperative day 0 | 66 | (8.7) |
| Postoperative day 1 | 484 | (63.8) |
| Postoperative day 2 | 95 | (12.5) |
| Postoperative day 3 | 42 | (5.5) |
| Later than postoperative day 3 | 72 | (9.5) |
| SGLT2i restart no. (%) |  |  |
| Postoperative day 0 | 3 | (0.4) |
| Postoperative day 1 | 167 | (21.9) |
| Postoperative day 2 | 118 | (15.5) |
| Postoperative day 3 | 72 | (9.5) |
| Later than postoperative day 3 | 235 | (30.9) |
| Not resumed | 164 | (21.6) |
| Ketoacidosis after postoperative day 3 no. (%) | 0 | (0.0) |
| Median duration of hospitalization after surgery day (IQR) | 9 | (6, 15) |

Percentages may not total 100 because of rounding. SGLT2i, sodium-glucose cotransporter 2 inhibitor

**Online Resource 5.** **Details of cases developed postoperative acidosis in whom urine ketone was negative, but BGA was performed as a routine postoperative examination**

| Res ID | Age | Sex | Surgery | Duration of anesthesia, min | SGLT2is | Urine ketone | Day of exam POD | pH | HCO_3_  mEq/L | PaCO_2_  Torr | AG  mEq/L | BG  mg/dL |
| --- | --- | --- | --- | --- | --- | --- | --- | --- | --- | --- | --- | --- |
| 099 | 70 | F | Abdominal (laparoscopic) | 206 | Dapagliflozin | Negative | 0 | 7.286 | 24.6 | 53.5 | 6.4 | 121 |
| 131 | 61 | M | Thoracoabdominal (esophagostomy) | 640 | Canagliflozin | Negative | 0 | 7.282 | 19.5 | 42.5 | 9.5 | 189 |
| 364 | 59 | F | Hysterectomy (laparoscopic) | 310 | Empagliflozin | Negative | 0 | 7.288 | 23.6 | 50.5 | 9.7 | 83 |
| 542 | 85 | F | Cardiovascular (valve replacement) | 234 | Canagliflozin | Negative | 0 | 7.290 | 21.2 | 47.9 | -1.2 | 152 |
| 612 | 61 | F | Abdominal (laparoscopic) | 339 | Empagliflozin | Negative | 0 | 7.295 | 18.4 | 38.1 | 9.6 | 161 |
| 685 | 76 | M | Abdominal (laparotomy) | 354 | Tofogliflozin | Negative | 0 | 7.286 | 21.8 | 46.9 | 4.0 | 134 |

AG, anion gap; BG, blood glucose; SGLT2is, sodium-glucose cotransporter 2 inhibitors

**Online Resource 6. Details of cases with urine ketone positive but missing blood gas analysis**

| Res ID | Age | Sex | Surgery | Duration of anesthesia, min | SGLT2i | ICU admission | Mechanical ventilation | Discharge  POD |
| --- | --- | --- | --- | --- | --- | --- | --- | --- |
| 44 | 59 | M | Orthopedic (lower extremity) | 157 | Ipragliflozin+sitagliptin* | no | no | 1 |
| 51 | 69 | M | Inguinal hernia repair | 173 | Dapagliflozin | no | no | 4 |
| 101 | 66 | F | Mastectomy | 124 | Ipragliflozin | no | no | 4 |
| 146 | 48 | F | Abdominal (laparotomy) | 192 | Empagliflozin | no | no | 5 |
| 148 | 59 | M | Orthopedic (upper extremity) | 172 | Luseogliflozin | no | no | 3 |
| 213 | 70 | M | Abdominal (laparoscopic) | 258 | Tofogliflozin | no | no | 8 |
| 228 | 81 | M | Cardiovascular (endovascular) | 174 | Empagliflozin | no | no | 8 |
| 235 | 55 | M | Proctectomy (laparoscopic) | 284 | Dapagliflozin | no | no | 8 |
| 241 | 45 | M | Adenoidectomy | 88 | Ipragliflozin | no | no | 7 |
| 242 | 88 | F | Cardiovascular | 259 | Empagliflozin | no | no | 10 |
| 243 | 59 | M | Orthopedic (extremity) | 178 | Ipragliflozin | no | no | 17 |
| 252 | 74 | F | ENT | 110 | Empagliflozin | no | no | 15 |
| 253 | 62 | F | Abdominal (laparoscopic) | 217 | Dapagliflozin | no | no | 9 |
| 270 | 44 | F | Eye | 124 | Empagliflozin+linagliptin* | no | no | 8 |
| 271 | 58 | M | Cholecystectomy (laparoscopic) | 156 | Empagliflozin | no | no | 6 |
| 273 | 57 | F | Transurethral urologic | 139 | Ipragliflozin | no | no | 7 |
| 275 | 63 | M | Thoracic (thoracoscopic) | 173 | Canagliflozin+teneligliptin* | no | no | 4 |
| 285 | 62 | M | Orthopedic (spine microsurgery) | 442 | Empagliflozin | no | no | 15 |
| 358 | 50 | M | Abdominal (percutaneous) | 103 | Empagliflozin | no | no | 6 |
| 426 | 47 | F | Hysterectomy (vaginal) | 111 | Ipragliflozin | no | no | 7 |
| 453 | 61 | M | Craniotomy | 221 | Tofogliflozin | no | no | 3 |
| 490 | 26 | M | Adenoidectomy | 185 | Dapagliflozin | no | no | 7 |
| 495 | 71 | F | Orthopedic (lower extremity) | 273 | Empagliflozin | no | no | 15 |
| 496 | 61 | M | Orthopedic (knee replacement) | 290 | Empagliflozin | no | no | 24 |
| 519 | 60 | F | Orthopedic (hip replacement) | 178 | Empagliflozin | no | no | 24 |
| 523 | 77 | M | Orthopedic (lower extremity) | 120 | Empagliflozin | no | no | 16 |
| 528 | 71 | F | Skin | 218 | Empagliflozin | no | no | 21 |
| 532 | 86 | F | Cardiovascular (endovascular) | 176 | Dapagliflozin | yes | no | 27 |
| 572 | 65 | F | Abdominal (laparotomy) | 370 | Empagliflozin | no | no | 11 |
| 577 | 65 | M | Orthopedic (lower extremity) | 355 | Dapagliflozin | no | no | 31 |
| 623 | 79 | F | Orthopedic (lower extremity) | 102 | Dapagliflozin | no | no | 2 |
| 677 | 51 | F | ENT | 166 | Empagliflozin | no | no | 6 |
| 754 | 63 | M | Orthopedic (lower extremity) | 207 | Dapagliflozin | no | no | 9 |
| 757 | 63 | M | Abdominal (laparoscopic) | 293 | Empagliflozin | no | no | 9 |

*Combination tablets. ENT, ear nose and throat; POD, postoperative day; SGLT2i, sodium-glucose cotransporter 2 inhibitors
